# Licensing Virus-Specific T Cells to Secrete the Neutrophil Attracting Chemokine CXCL-8 during Hepatitis B Virus Infection

**DOI:** 10.1371/journal.pone.0023330

**Published:** 2011-08-18

**Authors:** Adam J. Gehring, Sarene Koh, Adeline Chia, Komathi Paramasivam, Valerie Suk Peng Chew, Zi Zong Ho, Kang Hoe Lee, Mala K. Maini, Krishnakumar Madhavan, Seng Gee Lim, Antonio Bertoletti

**Affiliations:** 1 Singapore Institute for Clinical Sciences, Agency for Science Technology and Research (A*STAR), Singapore, Singapore; 2 Division of Clinical Microbiology, Department of Laboratory Medicine, Karolinska Institute, Stockholm, Sweden; 3 The Singapore Immunology Network, Agency for Science Technology and Research (A*STAR), Singapore, Singapore; 4 Asian Center for Liver Diseases and Transplantation, Gleneagles Hospital, Singapore, Singapore; 5 Division of Infection and Immunity, University College London, London, United Kingdom; 6 Department of Surgery, National University of Singapore, Singapore, Singapore; 7 Department of Medicine, National University of Singapore, Singapore, Singapore; Agency for Science, Technology and Research - Singapore Immunology Network, Singapore

## Abstract

T cell functional plasticity helps tailor antiviral immunity during different phases of infections. We tested whether, during different phases of HBV infection, virus-specific T cells can acquire specific proinflammatory functions that could drive granulocyte/mononuclear cell liver infiltration. Multifunctional analysis of HBV-specific T cells during acute and chronic HBV infection revealed that HBV-specific T cells had the capacity to produce the neutrophil chemokine CXCL-8 but not IL-17. CXCL-8 producing T cells were detectable in the liver of chronic HBV patients with active hepatitis; while in acute HBV patients CXCL-8 production by T cells was temporally limited to the acute phase of disease, concomitant with the peak of liver inflammation. Characterization of the conditions necessary for the development of CXCL-8 producing T cells showed a requirement for IL-7 and IL-15 during T cell expansion. These data show that functional plasticity of virus-specific T cells spontaneously occurs during HBV infection and that an environment rich IL-7 and IL-15 can license T cells with the ability to produce CXCL-8 and potentially influence liver pathology.

## Introduction

Virus-specific T cells are essential for the control of viral infections but are also implicated in triggering the inflammatory events that are often the main cause of pathology. This is particularly true during infection with non-cytopathic viruses such as hepatitis B virus (HBV), which causes acute and chronic liver inflammation; pathological conditions that can evolve into liver cirrhosis and hepatocellular carcinoma [1]. Virus-specific T cells are associated with control of HBV infection [2], [3]. Their frequency and function is far superior in patients who resolve HBV infection than in subjects with chronic infection and their ability to produce anti-viral cytokines (IFN-γ and TNF-α) results in HBV clearance from infected hepatocytes without extensive direct killing [4], [5], [6], [7], [8].

In addition to their protective role, intrahepatic activation of HBV-specific T cells can also trigger an influx of inflammatory granulocytes and mononuclear cells to the liver, which is the principle cause of hepatic injury. This has been clearly demonstrated in experimentally infected chimpanzees and in HBV transgenic mice. Depletion of T cells in infected chimpanzees reduced liver inflammation but prolonged HBV infection [2], [3]. In mice, adoptively transferred HBV-specific T cells trigger the recruitment of neutrophils and mononuclear cells that result in liver damage [9]. Notably, depletion of neutrophils prior to T cell transfer abolished the inflammatory infiltrate without hindering the antiviral efficiency of HBV-specific T cells or reducing CXCL-9 and CXCL-10 production, two chemokines induced by IFN-γ known to recruit inflammatory cells to the liver [10], [11].

The ability of virus-specific T cells to orchestrate such inflammatory phenomenon is generally ill defined in different human pathologies. Thus, the goal of the present study was to characterize the inflammatory potential of virus-specific T cells, analyzing their ability to produce different effector molecules in a non-cytopathic human infection such as HBV. Since the role of interferon inducible chemokines have already been investigated [12] we focused our attention on IL-17 and CXCL-8 due to their inflammatory potential and ability to recruit neutrophils, which represents a key step in animal models of acute viral hepatitis. CXCL-8, which is the primary chemotactic factor for neutrophils, is a less well characterized T cell derived chemokine but can be produced in large quantities by T cells [13], [14] and elevated levels of CXCL-8 are found in patients with chronic liver disease [15] and chronic HBV patients prior to hepatic flares [16]. Likewise, IL-17 is known to recruit neutrophils [17] and has been associated with inflammatory diseases [18], [19], including hepatic flares in chronic HBV patients [20].

Since T cells display a degree of functional plasticity, the environment where T cells are activated can have a dramatic effect on their ultimate function [21], [22]. Thus, we hypothesized that the inflammatory cytokine milieu present during HBV infection can license T cells with the ability to produce CXCL-8 or IL-17. IL-15 is elevated in the liver of patients with active hepatitis [23], [24], has been demonstrated to induce IL-17 production [25], [26] and can stimulate CXCL-8 and MCP-1 expression from monocytes [27]. In addition, IL-7 can be up-regulated in the liver by inflammation and enhances T cell cytotoxic activity and cytokine production [28]. Therefore, we focused on these two cytokines, which are present in the liver during inflammation, and are known to impact T cell function.

Our data demonstrate that HBV-specific T cells produce CXCL-8, but not IL-17, during periods of liver inflammation and that this functional phenotype could be induced in greater than 90% of the detectable virus-specific T cell population in acute/resolved HBV patients by exposure to IL-7 and IL-15. We characterized the phenotype and functional profile of CXCL-8+ T cells and demonstrated that this functional profile could be induced in unrelated CMV-specific T cells from healthy individuals. Thus, cytokines present during inflammation, particularly in the liver, could license T cells with additional cytokine profiles that contribute to tissue inflammation.

## Results

### HBV-specific T cell related chemokines

To begin investigating the potential contribution of additional T cell functional profiles to liver inflammation, outside of IFN-γ induced CXCL-9 and CXCL-10, we analyzed peptide-specific inflammatory cytokine/chemokine production from liver resident lymphocytes of HBV patients. We tested intrahepatic T cell responses to pools of 15mer overlapping peptides covering all HBV proteins from six chronic active hepatitis B patients that developed hepatocellular carcinoma (HCC). Intrahepatic lymphocytes, isolated from non-tumor tissue, were stimulated with peptides overnight and culture supernatants were screened for CXCL-8, CXCL-9, CXCL-10, CCL-5, CCL-2 as well as IL-17 and the anti-inflammatory cytokine IL-10.

**Figure 1:**
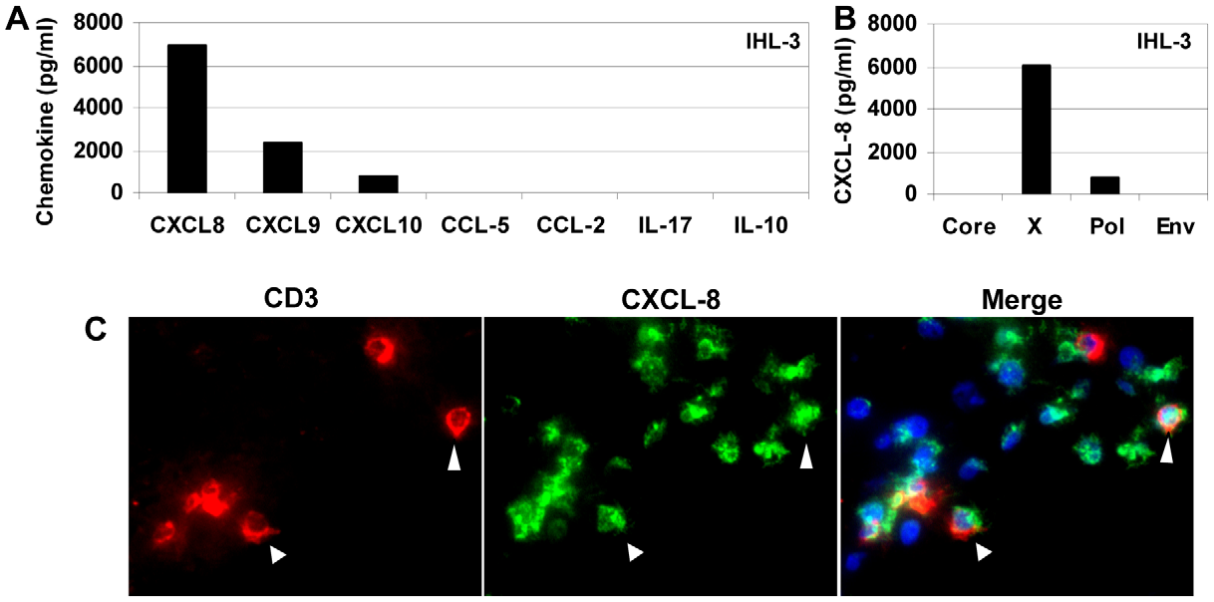
Chemokine production following HBV peptide stimulation of intrahepatic mononuclear cells. **A**) Cumulative amount of chemokines produced in response to all HBV peptides in a representative HBV patient. **B**) Antigen distribution of CXCL-8 production from panel A. All supernatants were harvested after 20 h culture and background from unstimulated wells was subtracted to give peptide specific concentrations. **C**) Representative immunofluorescence staining of a biopsy from a chronic HBV patient with elevated ALT. Figure shows CD3 (red, left panels), CXCL-8 (green, middle panels) and merged images, including DAPI staining (right panels).

Unlike resolved HBV patients that display a multi-specific T cell response, T cells from chronic HBV patients are often exhausted or deleted due to the persistent nature of the infection; thus, we were only able to detect peptide-specific responses in two of the patients tested. The profile of one patient (IHL-3) is represented in figure 1A. CXCL-8 was the dominant inflammatory mediator detected, followed by the IFN-γ inducible chemokines CXCL-9 and CXCL-10. Patient IHL-1 showed polymerase specific production of CXCL-8 but CXCL-9 and CXCL-10 were undetectable (data not shown). We did not detect peptide-specific production of IL-17 or IL-10 in any sample and only low levels were observed following CD3/CD28 stimulation (Fig. 1A and data not shown). Figure 1B shows the antigen-specific distribution of CXCL-8 production in patient IHL-3, with a majority of the CXCL-8 response specific to the HBV X peptide pool. CXCL-9 and CXCL-10 production in IHL-3 followed an identical antigen-specific distribution (data not shown). This suggests that there are liver resident, HBV-specific T cells capable of responding to HBV antigens by producing CXCL-8.

The limited number of cells and responses precluded any firm conclusions since CXCL-8 can also be produced by macrophages, Kupffer cells, granulocytes as well as hepatocytes. Therefore, we used immuno-fluorescence to confirm that CXCL-8 producing T cells are present in the liver of chronic HBV patients. Biopsies from four chronic HBV patients were stained with anti-CD3 and anti-CXCL-8. Multiple CD3+/CXCL-8+ cells were detected in two separate patients in multiple fields, confirming the presence of CXCL-8 producing T cells in the liver of chronic HBV patients (Fig. 1C).

### No IL-17 production by HBV specific T cells

The absence of peptide-specific IL-17 production in the intrahepatic samples may be due to the relatively limited number of response detected overall or could be related to the fact that the intrahepatic samples had relatively little inflammation (ALT <150 U/L for all samples). To further investigate the possibility of virus-specific IL-17 producing T cells we tested acute HBV patients (5 ex vivo, 4 in vitro expanded) with serum levels of alanine-aminotransferase (ALT), an enzyme released upon hepatocyte damage, greater than 1000 U/L, chronic HBV patients (6 ex vivo, 5 in vitro expanded) with elevated ALT (5/6 patient ALT >1000 U/L) and intrahepatic lymphocytes isolated from chronic HBV patients (4 ex vivo) by IL-17-specific Elispot using overlapping peptides covering the entire HBV proteome. Some chronic HBV patients had elevated ALT at multiple time points and thus were tested repeatedly (ex vivo n = 11; in vitro n = 9).

**Figure 2:**
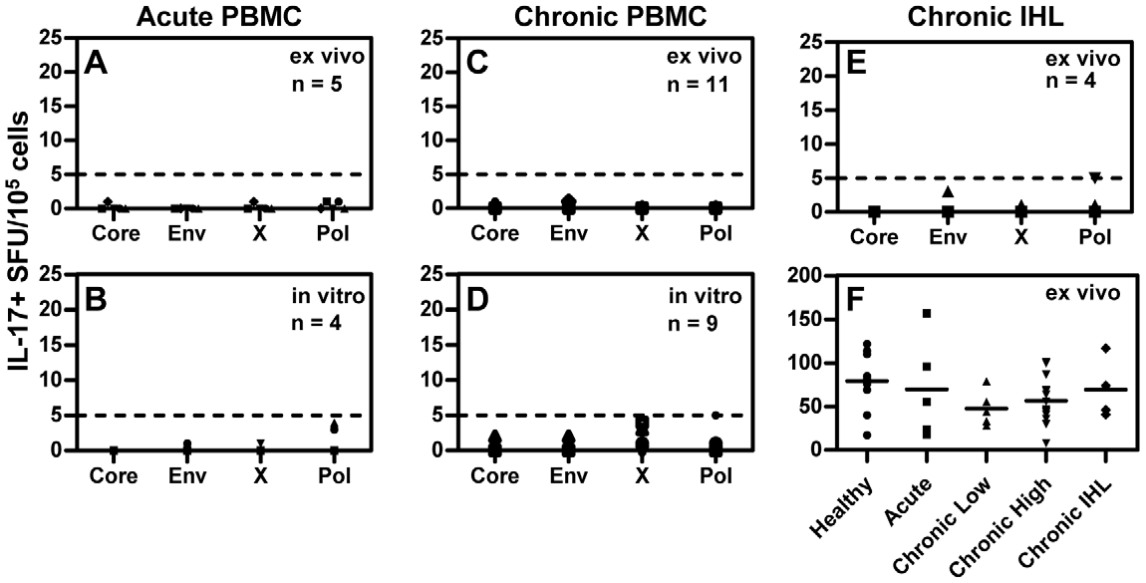
No IL-17 producing T cells were found in HBV patients. Peptide-specific IL-17 elispot was performed using PBMC from acute HBV patients (**A**) ex vivo or (**B**) after in vitro expansion and on chronic HBV patients with elevated ALT (**C**) ex vivo or (**D**) after in vitro expansion and on (**E**) IHL from chronic HBV patients. **F**) Ex vivo stimulation with SEB to determine if the frequency of non-specific IL-17 producing cells was increased in patients with active liver inflammation. All data expressed as spot forming units (SFU)/10^5^ cells.

**Figure 3:**
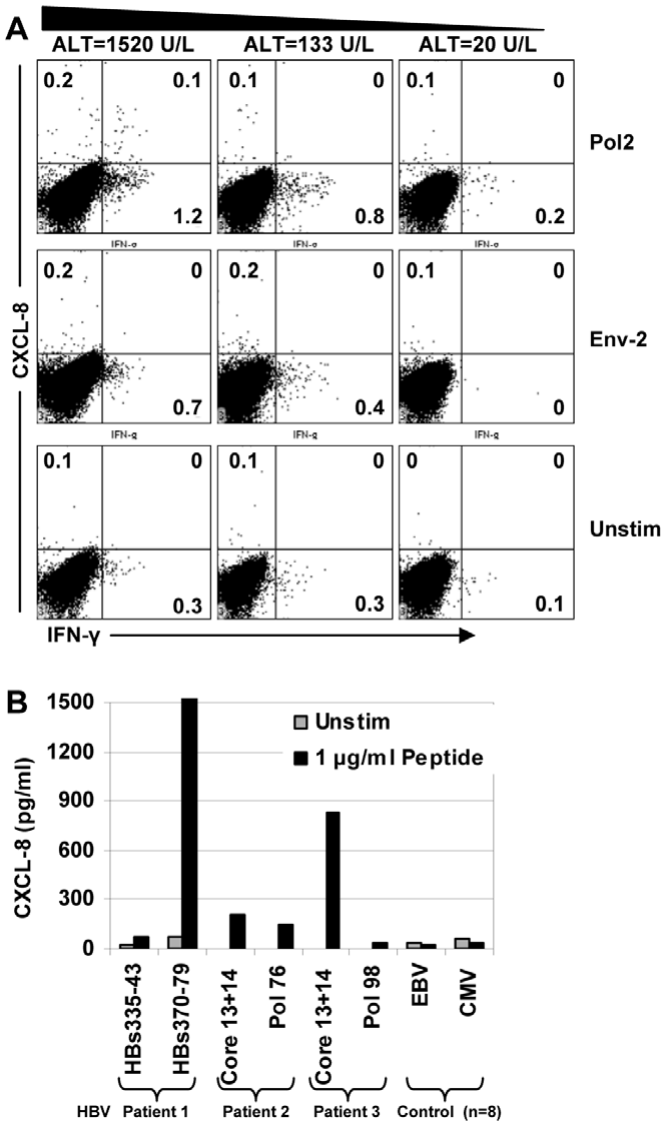
T cell CXCL-8 production in acute HBV patients. **A**) Longitudinal PBMC samples tested with pools of HBV peptides for specific IFN-γ and CXCL-8 production; Pol-2 peptide pool (top row) produced CXCL-8, Env-2 (Middle row) produced only IFN-γ, unstimulated cells (bottom row) show the background at each time point. Dot plots were gated on total T cells. **B**) CXCL-8 produced from short-term T cell lines expanded from acute HBV patients +/− 1 µg/ml peptide for 20 h. Multiple responses for three separate patients are displayed. As negative control, EBV and CMV-specific short-term T cell lines were expanded from 8 healthy donors and analyzed for CXCL-8 production. The mean CXCL-8 production is shown for the healthy donors.

**Figure 4:**
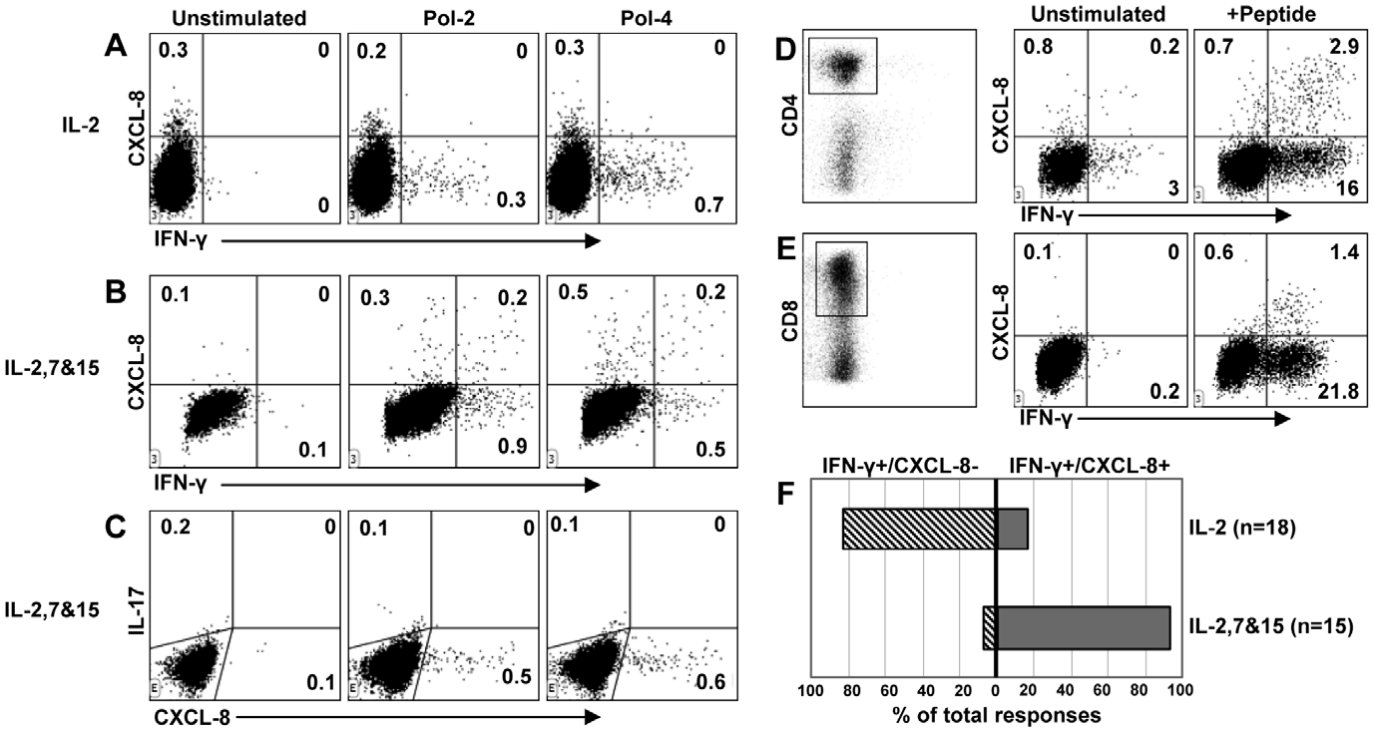
IL-7 and IL-15 induce CXCL-8 production. Acute patient PBMC cultured in **A**) IL-2 alone or **B**) IL-2/IL-7/IL-15 were expanded with peptides covering the entire HBV proteome and tested with peptide pools for IFN-γ and CXCL-8 production. **C**) PBMC from the same patient expanded in IL-2/IL-7/IL-15 were tested with peptide pools for IL-17 and CXCL-8 production. **D**) HBV-specific CD4 T cells and **E**) HBV-specific CD8 T cells restimulated in IL-2/IL-7/IL-15 expand and maintain CXCL-8 production. **F**) Distribution of functional phenotypes of all HBV-specific T cell responses detected in acute/resolved HBV patients after in vitro culture in IL-2 or IL-2/IL-7/IL-15.

We did not detect any HBV-specific IL-17 producing T cells in acute or chronic HBV patients ex vivo or after in vitro expansion (Fig. 2 A–E). Following SEB stimulation, we did not observe any increase in non-specific IL-17+ T cells in acute HBV patients with high ALT (>1000 U/L), chronic HBV patients with normal ALT (<50 U/L), chronic HBV patients with raised ALT (>100 U/L) or intrahepatic lymphocytes from chronic HBV patients compared to that observed in healthy donors (Fig. 2F). Virus-specific cells were present when screened for IFN-γ (Fig. 3 & 4 and data not shown) indicating that HBV-specific T cells are present but do not produce IL-17.

### CXCL-8 producing T cells in HBV patients

There is no CXCL-8 Elispot assay available and since CXCL-8 can be produced by many different cell types we tested T cell CXCL-8 production using flow cytometry to be certain production was from T cells. We first tested 3 acute HBV patients that had longitudinal samples from onset to resolution. T cells were expanded for 10 d using overlapping peptides and tested for IFN-γ and CXCL-8 production. IFN-γ responses were detected to peptides pools in all three patients. Two patients had small but detectable populations of CXCL-8+/IFN-γ+ T cell responses at the onset of disease; one representative patient is shown in figure 3A. CXCL-8+ T cells were only detectable to the polymerase Pol-2 peptide pool at the onset of disease (ALT = 1520 U/L) and disappeared as liver inflammation subsided, whereas IFN-γ+ responses remained detectable (Fig. 3A, top row). CXCL-8 producing T cells comprised only a portion of the IFN-γ+ response and were not detectable in other responses within the same patient (Env-2; Fig. 3A, middle row).

Since the frequency of CXCL-8+ T cells was small we tested short-term T cell lines from 3 additional acute HBV patients, where we identified individual peptide responses using IFN-γ Elispot (Figure S1), for peptide specific CXCL-8 production. T cell lines from acute patients were stimulated with the indicated peptides and CXCL-8 was measured in the supernatant after overnight culture. CXCL-8 production was variable between patients, and between responses within the same patient, but was clearly detectable in response to peptide stimulation, whereas EBV and CMV specific T cells expanded from 8 healthy donors were all negative (Fig. 3B). These data confirm that virus-specific T cells have the capacity to produce CXCL-8 but it was unclear whether CXCL-8 production was an inducible function or represented a distinct lineage that became undetectable as the T cell response contracted with disease resolution.

### IL-7 and IL-15 induce CXCL-8 production in HBV-specific T cells

To determine if CXCL-8 production was an inducible phenotype and, as hypothesized above, if exposure to IL-7 and IL-15 could play a role we expanded PBMC from 6 acute/resolved HBV patients in the presence of IL-2 alone or IL-2 plus IL-7 and IL-15 and tested for HBV-specific CXCL-8 producing T cells. T cells grown in IL-2 alone produced IFN-γ but little or no CXCL-8 (Fig. 4A). In contrast, cells from the same patient, grown in IL-2+IL-7+IL-15 in parallel, showed a significant increase in CXCL-8 producing T cells (Fig. 4B). Unlike before when CXCL-8 production was not observed in multiple responses within the same patient and barely detectable after peptide stimulation, CXCL-8 production was much greater and a CXCL-8+ population could be detected in all the IFN-γ+ responses from this patient. In addition to IFN-γ+/CXCL-8+ T cells, we also observed a population of CXCL-8 single positive T cells. We also examined whether IL-7 and IL-15 induced the production of IL-17 in these cells but as demonstrated in figure 4C, even after in vitro expansion in IL-7 and IL-15 HBV-specific T cells did not produce IL-17. We were also able to further expand these cells in vitro and demonstrate that this functional phenotype could be induced/maintained in both CD4 (Fig. 4D) and CD8 (Fig. 4E) T cells.

Overall, for T cells expanded in IL-2 alone we detected 18 IFN-γ+ T cell responses distributed between all four HBV proteins (Table 1). Of the 18 IFN-γ+ responses, only three were IFN-γ+/CXCL-8+ (Table 1). In contrast, when T cells were expanded in the presence of IL-7 and IL-15 we found that 14/15 (93%) of the virus-specific responses detected were IFN-γ+/CXCL-8+ (Table 1 and Fig. 4F). The culture conditions clearly altered the function of HBV-specific CD8 and CD4 T cells and induced the ability to produce CXCL-8. Even if cells were first expanded in IL-2 alone, further stimulation in medium containing IL-2, IL-7 and IL-15 could induce this functional alteration (data not shown). When antigen specific distribution of IFN-γ+/CXCL-8+ T cells was analyzed, we found that they were evenly distributed between the different HBV proteins, similar to what was found in cells cultured in IL-2 alone (Table 1). Thus, IFN-γ/CXCL-8 producing virus-specific T cells can be induced to encompass almost the entire population of HBV-specific T cells given the appropriate conditions.

**Table 1:** Frequency and cytokine profile of T cell responses from acute HBV patients.

Patient	Acute/Resolved			
Culture Condition	IL-2		IL-2+IL-7+IL-15	
cytokine profile^a^	IFN-γ+	γ+/8+	IFN-γ+	γ+/8+
# responses^b^	15/18	3/18	1/15	14/15
HBV antigen				
Core	3	0	0	5
Env	4	1	1	4
Pol	7	2	0	5
X	1	0	0	0

a Type of cytokine response detected IFN-γ+ = IFN-γ+/CXCL-8−; γ+/8+ = IFN-γ+/CXCL-8+.

b number of responses corresponding to cytokine profile out of total IFN-γ+ responses detected.

### Characterization of CXCL-8 producing T cells

**Figure 5:**
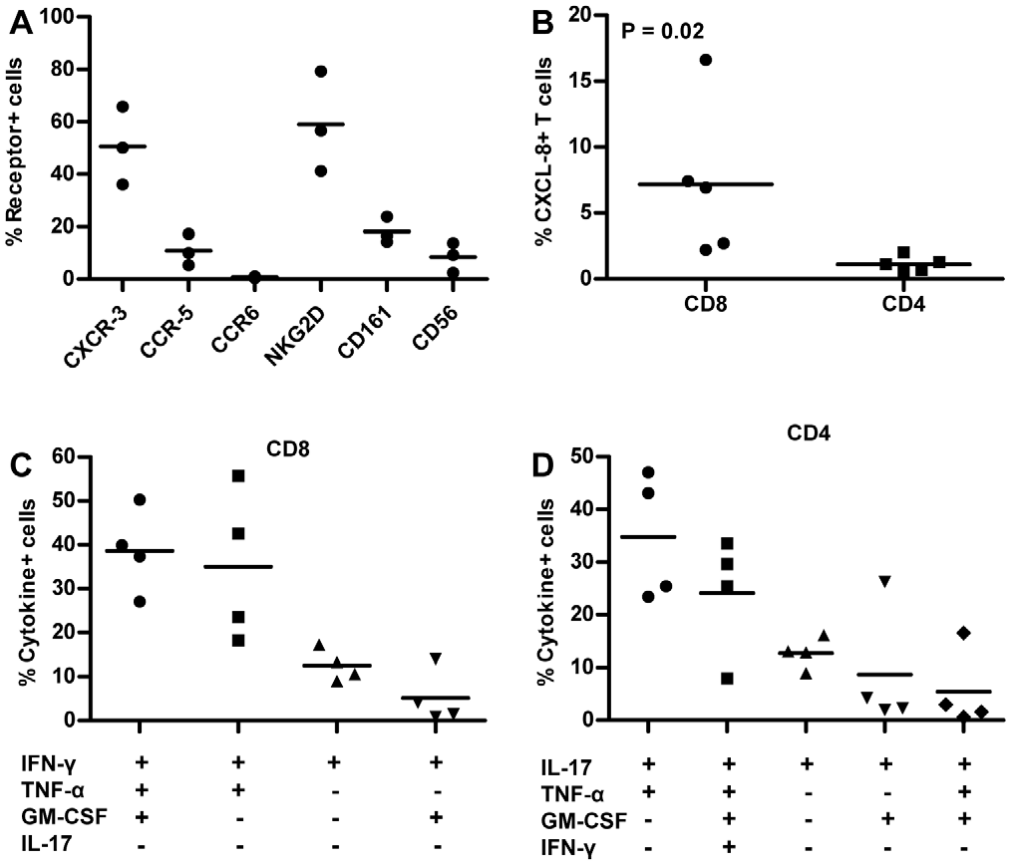
Characterization of CXCL-8+ T cells. **A**) Receptor expression on CXCL-8+ T cells from healthy donor PBMC expanded for 7 d in IL-2/IL-7/IL-15. Graph shows the frequency of receptor expression on total CXCL-8+ T cells from three donors. **B**) Frequency of CD8 and CD4 CXCL-8+ T cells in total T cell population from 5 healthy donors after in vitro expansion. **C**) Functional profile of **C**) CXCL-8+/CD8+ T cells and **D**) CXCL-8+/CD4+ T cells. Shown are frequencies of cytokine profiles within the CD8/CXCL-8 or CD4/CXCL-8 from 4 healthy donors.

To characterize the phenotype of CXCL-8 producing T cells we stained for a panel of receptors that are associated with intrahepatic T cells in HBV patients and CCR6, which is generally found on IL-17 producing T cells. We expanded PBMC from healthy donors for 7 d in IL-2, IL-7 & IL-15 using anti-CD3 and examined receptor expression on CXCL-8+ T cells after mitogen stimulation. We found that the dominant population within the CXCL-8+/CD3+ T cells expressed CXCR3 and NKG2D (Fig. 5A). CCR5, CD161 and CD56 were also expressed on 10–20% of CXCL-8+ T cells whereas CCR6 expression was low.

To characterize their function we first determined which T cell subset had the highest frequency of CXCL-8 producing T cells. As seen in figure 5B, CXCL-8+ T cell frequency was significantly higher in CD8 T cells compared to CD4 T cells. We then analyzed the cytokine profile of CD8+/CXCL-8+ and CD4+/CXCL-8+ T cells using polychromatic flow cytometry. CXCL-8+ T cells were multifunctional but the cytokine profiles differed between the CD8 and CD4 T cells. The largest population within the CD8 T cells (40% of CXCL-8+/CD8+ T cells) produced a total of four cytokines CXCL-8, IFN-γ, TNF-α, and GM-CSF (Fig. 5C). CD8 T cells producing CXCL-8, IFN-γ and TNF-α represented a similar frequency (Fig. 5C). In contrast, the largest population in the CD4 T cells produced CXCL-8, IL-17, and TNF-α (35% of CXCL-8+/CD4+ T cells) followed by cells that were capable of producing 5 different cytokines (Fig. 5D). The dominant cytokine within the CXCL-8+/CD4+ T cells was IL-17, suggesting that Th17 cells may produce CXCL-8.

### Inducible CXCL-8 production in unrelated virus-specific T cells

**Figure 6:**
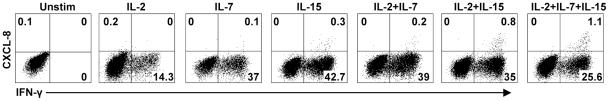
Induction of CXCL-8 in CMV-specific CD8 T cells cultured for 10 d in the cytokine indicated above respective plots. Following expansion, CMV-specific cells were stimulated with 1 µg/ml peptide and stained for CXCL-8 and IFN-γ.

We determined if CXCL-8 production was unique to HBV-specific T cells by testing the culture conditions (IL-2, IL-7 and IL-15) on memory T cells specific for an unrelated virus. CMV-specific T cells from healthy donors were expanded in different combinations of IL-2, IL-7 and IL-15 and we monitored their ability to produce IFN-γ and CXCL-8 by intracellular cytokine staining. Similar to HBV-specific T cells, culture in IL-2 alone yielded IFN-γ+ T cells but did not induce CXCL-8 production; neither did IL-7 and IL-15 only weakly induced CXCL-8 production by CMV specific cells (Fig. 6). The combination of IL-2/IL-7 did not boost CXCL-8 production over the individual cytokines but the combination of IL-2 and IL-15 did increase CXCL-8+ T cell frequency. However, culture in all three cytokines induced the most robust CXCL-8 production by CMV-specific T cells (Fig. 6). Detection of CXCL-8+ T cells was not simply a consequence of detection limit. T cells cultured in IL-7 alone, IL-15 alone and IL-2+IL-7 resulted in much higher frequencies of IFN-γ+ T cells (35–42%) but low/undetectable frequencies of CXCL-8+ T cells compared to cells grown in IL-2+IL-7+IL-15 (25% IFN-γ+ and 1.1% CXCL-8+) These data indicate that CXCL-8 production is not restricted to HBV-specific T cells and is an inducible functional phenotype that can impact other virus-specific T cell populations.

## Discussion

Recent reports have demonstrated the functional plasticity of memory T cells, where at least a small proportion of the memory cell population can be reprogrammed based on environmental queues [22]. This flexibility in T cell function likely helps tailor the antiviral T cell response to the site of infection. In our study, we examined virus-specific T cell function in the liver of chronic HBV patients and longitudinally in patients during the changing environment from disease onset to resolution. We identified a population of HBV-specific T cells able to produce CXCL-8 in the setting of liver inflammation; however, this function disappeared as inflammation resolved in acute patients. Using cytokines that have been identified in the inflammatory liver environment we were able to re-induce CXCL-8 production not only in HBV-specific responses but also unrelated CMV-specific responses from healthy donors.

CXCL-8 production by T cells was previously a rare quality. Examples of CXCL-8 producing T cells have only been described in immune-mediated inflammatory skin reactions such as drug-specific acute generalized exanthematous pustulosis (AGEP) and to our knowledge there has been no such description of this function in pathogen-specific T cells [13], [14], [29]. Despite the lack of previous examples, we detected peptide-specific CXCL-8 production from intrahepatic lymphocytes of chronic HBV patients and from peripheral blood T cells in acute/resolved patients. After culturing acute/resolved HBV patient PBMC in IL-7 and IL-15 we could detect CXCL-8 producing T cells in the majority of HBV-specific responses. We attempted to use surface markers to identify CXCL-8 producing T cells and delineate whether IL-7 and IL-15 were inducing a unique population of virus-specific T cells or altering the function of classical memory T cells; however, we were unable to ascertain a distinct phenotype to answer this question. We did observe that if cells were first cultured in IL-2 alone for 10 d, further in vitro expansion in the presence of IL-7 and IL-15 could induce CXCL-8 production (data not shown), suggesting that these cytokines are altering T cell function.

The fact that IL-7 and IL-15 were required to detect CXCL-8 producing T cells in nearly all HBV-specific responses suggests a particular environment is necessary before virus-specific T cells are licensed with such inflammatory function. IL-15 has been associated with multiple inflammatory diseases [25], [30], [31], is up-regulated following liver injury [32] and is increased in patients with chronic hepatitis B and C infection [23], [24], [33]. Furthermore, IL-15 can be produced by hepatic stellate cells (Ito cells) [34], a specialized liver-resident antigen presenting cell, while IL-7 can be produced by hepatocytes [28]. Thus, it is possible that the liver is a particularly well suited environment for the induction of CXCL-8 producing virus-specific T cells. However, it was clear from our experiments with CMV-specific T cells that induction of CXCL-8 production is not limited to HBV-specific T cells and IL-7 and IL-15 can alter the functional profile of memory T cells to unrelated viruses in healthy individuals.

There appeared to be lineage differences between CXCL-8+ CD8 and CD4 T cells. While a large proportion of the CXCL-8+ T cells expressed surface receptors consistent with a Th1/Tc1 profile, which was evident in the functional profile of CXCL-8+/CD8+ T cells, a majority of the CXCL-8+/CD4+ T cells produced IL-17. Chemokine receptor expression on CXCL-8+ T cells was not differentiated into CD4/CD8 but CXCL-8+ CD4 T cells represented a relatively small fraction of the total T cell population, which was consistent with the low frequency of CCR6 detected on total CXCL-8+ T cells. However, it is important to note that this functional phenotype, which was observed after PMA/ionomycin stimulation, was not detected in any of the antigen specific assays in our study.

We tested extensively for the presence of virus-specific IL-17 producing T cells in all of the assays, ex vivo and after in vitro expansion, but were unable to find a significant HBV-specific population by Elispot, intracellular cytokine staining or peptide specific production in the supernatant of intrahepatic lymphocytes. In addition to the lack of HBV-specific IL-17 production, we did not observe an increase in non-specific IL-17 producing T cells using Elispot after SEB stimulation in acute and chronic HBV patients. We previously characterized cytokines detectable in the serum of HBV patients and were unable to detect IL-1β or IL-6, two cytokines that play a role in the development of Th17 cells [12]. Therefore, the inflammatory environment during HBV infection may not lend itself to Th17 differentiation. However, our data on non-specific IL-17 production is in contrast to recent reports suggesting that IL-17 producing T cells were increased in chronic HBV patients with liver inflammation [20]. This discrepancy could be due to the sample size or assays and mitogens used to stimulate IL-17 producing T cells. Additional studies, particularly in the intrahepatic compartment, will be necessary to determine if IL-17 producing cells are involved in HBV pathology but our data suggest that HBV-specific IL-17 producing T cells are not present in acute or chronic HBV patients.

Overall, the goal of the current study was to characterize a potential inflammatory profile that could exacerbate tissue and organ inflammation in non-cytopathic viral infections. The exact role of CXCL-8 in human liver inflammation is still not clear and whether CXCL-8 producing T cells actually contribute to or initiate the inflammatory process is an open question. However, our data demonstrate that human virus-specific T cells have, or can acquire through exposure to environmental factors, a cytokine/chemokine profile capable of contributing to parenchymal inflammation observed in non-cytopathic viral infection [9], [10], [11], [35].

## Materials and Methods

### Ethics Statement

This study was approved by the National University Hospital of Singapore Institutional review board and the Singapore Ethics Committee. Written informed consent was obtained from all subjects.

### Patients

Blood and liver specimens were collected with informed consent from 30 patients (Blood from 12 acute/resolved HBV patients, PBMC from 6 chronic patients plus 8 intrahepatic lymphocytes and 4 biopsies from chronic patients) infected with HBV from the Gleneagles Hospital and National University Hospital Singapore. Eight subjects had clinical, biochemical and virological evidence of acute hepatitis B infection (ALT levels >10 times the upper limits of normal, detection of HBsAg and serum anti-HBc IgM and HBsAg clearance within 2 months from clinical onset of hepatitis). PBMC from four patients were collected during the resolved phase following acute HBV infection. The 18 chronic hepatitis B patients studied had clinical, biochemical and virological evidence of chronic HBV infection (HBV-DNA+, HBsAg+, HBeAg+, and elevated levels of ALT).

All studied patients were negative for antibodies to hepatitis C virus (HCV) and delta virus. Virological assessment, HBsAg, HBeAg, anti-HBs, anti-HBc IgG and IgM, anti-HBe, anti-HDV, anti-HCV, were determined by commercial enzyme immunoassay kits (Abbott Labs, IL, USA; Ortho Clinical Diagnostic, Johnson & Johnson). Serum HBV-DNA was quantified by PCR (Cobas Amplicor test; Roche Diagnostic, Basel, Switzerland).

### Cell lines

Short-term T cell lines were grown for 10 d in AIM-V+2% human AB serum (Invitrogen, Carlsbad, CA) supplemented with 20 U/ml IL-2+/−10 ng/ml IL-7 and IL-15 (R&D systems). Briefly, PBMC were thawed and 20% of cells were pulsed with 10 µg/ml 15mer overlapping peptides spanning the entire HBV proteome (Mimotopes, Clayton, Victoria) for 1 h at 37°C, washed and mixed with remaining PBMC in 1 ml of medium and grown in 24 well plates. After 10 d expansion cells were tested using Elispot and intracellular cytokine staining with pools of overlapping peptides (1 core, 1 X, 2 envelope, 4 polymerase pools). For defined epitopes, 2.5×10^5^ cells/well on 96 well round bottom plates were stimulated with 1 µg/ml peptide and expanded in the media described above. To determine the surface and functional phenotype of CXCL-8 producing T cells, PBMC of healthy donors were expanded with anti-CD3 (OKT-3, eBioscience)+20 U/ml IL-2+10 ng/ml IL-7 and IL-15 for 7 d.

### Peptides

15mer peptides, overlapping by 10 amino acids, covering the entire HBV proteome were based on the HBV genotype C (GenBank: AB112063) and D (GenBank: AF121241) sequences (Mimotopes). Overlapping peptides were pooled according tor their protein and included up to 45 individual peptides; 1 core, 1 X, 2 envelope (Env), and 4 polymerase (Pol) pools were made. Defined amino acid epitopes HBV surface (HBs) 335-43 (WLSLLVPFV); HBs370-79 (SIVSPFIPLL); CMVpp65 495–504 (NLVPMVATV) were purchased from Genscript (Piscataway, NJ). Additional individual peptide responses were identified via IFN-γ Elispot screening (data not shown).

### Enzyme-Linked Immunosorbent Spot Assay (ELISPOT)

ELISPOT assays were performed as previously described using peptides covering the entire proteome of HBVgenC or HBVgenD [6]. T cell responses were analyzed directly *ex vivo* or after 10 d *in vitro* expansion with 1×10^5^ cells/well. Briefly, 96-well plates (Multiscreen-HTS; Millipore, Billerica, MA) were coated overnight at 4°C with 2.5 µg/ml capture mouse anti-human IL-17 antibody (eBio64CAP17; eBioscience). The plates were then washed with PBS, blocked and a total of 1×10^5^ cells were added to each well. HBV peptides from the patients' respective genotype were added to a final concentration of 2 µg/ml and plates were incubated for 18 hours at 37°C. Following the incubation, IL-17 spot forming units (SFU) were detected using 0.25 µg/ml anti-human IL-17 MAb (eBio64DEC17; eBioscience) followed by incubation with streptavidin-alkaline phosphatase (Mabtech, Sweden). The plates were washed and 50 µl of alkaline phosphatase substrate (5-bromo-4-chloro-3-indolyl phosphate–nitro blue tetrazolium chloride [BCIP-NBT]; KPL, Gaithersburg, MD) was added. Two wells were left without peptide as negative controls. Positive control was 10 µg/ml staphylococcal enterotoxin B (SEB; Sigma-Aldrich, St. Louis, MO). IL-17 secreting cells were expressed as (SFU) per 1×10^5^ cells. Assays were considered positive when SFU was above 5.

### Flow cytometry

For intracellular cytokine staining short term T cell lines were stimulated with 1–5 µg/ml of defined epitopes or overlapping peptide pools covering Core, X, envelope, polymerase for 6 h in the presence of 10 µg/ml brefeldin A (Sigma-Aldrich, St. Louis, MO). Following incubation, cells were surface labeled with CD8-PeCy7 or CD4-PeCy7 (BD Biosciences, San Jose, CA) and fixed using Cytofix/Cytoperm (BD Biosciences). Cells were stained with anti-IFN-γ-APC, TNF-α-Alexa488, anti-CXCL-8-PE (BD Biosciences) or anti-IL-17-Alexa488 (eBioscience) for 30 min on ice, washed and fixed in 1% formaldehyde. Data acquisition was performed using a BD FACs Canto flow cytometer.

T cells from healthy donors expanded in IL-2, IL-7 and IL-15 were stimulated with PMA/ionomycine for 6 h in the presence of 10 µg/ml brefeldin A after 7 d in vitro culture. Following stimulation cells were stained with CXCR3-APC, CCR5-APC, NKG2D-APC, CD161-APC, CD56-PeCy7 (BD Biosciences) or CCR6-APC (R&D Systems) and then fixed in cytofix/cytoperm. After fixation T cells were stained with anti-CXCL-8-PE. CXCL-8+ T cells were gated and analyzed for surface receptor expression. Isotype control antibodies were used as negative control to define positive populations. To examine the functional profile of CXCL-8+ T cells we stimulated T cells from healthy donors expanded in IL-2, IL-7 and IL-15 with PMA/ionomycin for 6 h in the presence of 10 µg/ml brefeldin A. T cells were labeled with anti-CD3-Q605 (Invitrogen), anti-CD8-APC-Cy7 and anti-CD4-PerCp (BD Biosciences) and fixed using cytofix/cytoperm. T cells were stained with anti-CXCL-8-PE, anti-IFN-γ-V450, anti-TNF-α-PeCy7 (BD Biosciences), anti-IL-17-Alexa488 and anti-GM-CSF-Alexa647 (eBioscience). Samples were acquired on BD LSR-II flow cytometer and data was analyzed in FACs Diva software.

### Chemokine/Cytokine measurement

Cell culture supernatants were tested for chemokine production, IL-17A and IL-10 using the Cytometric bead assay (BD Biosciences). Supernatants were collected after 20 h from 10^5^ cells (intrahepatic lymphocytes or short-term T cell line) +/− stimulation by individual peptides or peptide pools. Chemokines tested were CXCL-8 (IL-8), CCL-5 (RANTES), CXCL-9 (MIG), CCL-2 (MCP-1) and CXCL-10 (IP-10).

### Isolation of intrahepatic lymphocytes

Intrahepatic lymphocytes were isolated from liver samples collected from hepatic resections of hepatocellular carcinoma nodules from chronically infected HBV patients. Non-tumor tissue surrounding HCC were used to isolate intrahepatic lymphocytes. Tissue sections were dissociated by mechanical disruption using scalpels and then using an Ultra-Turrax tube drive (IKA, Wilmington, NC). Samples were digested with 1 U/ml Liberase blendzyme 3 (Roche, Basel, Switzerland) for 15 min at 37C with stirring. Samples were washed with HBSS centrifuged at 400×g for 10 min to remove Liberase enzyme. Tissue homogenate was re-suspended in HBSS and large debris and hepatocytes were separated by differential centrifugation at 85×g for 2 min. Differential centrifugation was repeated twice and supernatants taken from differential centrifugation were pelleted at 400×g for 10 min. The tissue sample pellet was re-suspended in RPMI and depleted of large debris by passage through a 100 µm vacuum driven filter (Millipore, Billerica, MA). Filtered samples were layered over Ficoll-Paque Plus (GE Healthcare, Piscataway, NJ) and intrahepatic lymphocytes were isolated by density gradient centrifugation.

### Immunofluorescent staining

Patient biopsies were thawed, dried at room temperature and fixed with acetone. Slides were blocked with 10% normal goat Serum (DAKO) for 30 min at room temperature then incubated with mouse monoclonal anti-human CXCL-8 (BD Pharmigen; Clone G265-8) in combination with rabbit anti-human CD3 (DAKO) followed by AlexaFluor594-conjugated anti-rabbit and AlexaFluor488-conjugated anti-mouse Abs (Invitrogen). Nuclei were stained with DAPI (Invitrogen). Images were acquired using Olympus IX 81 inverted microscope and ImagePro software.

## Supporting Information

Figure S1 T cell responses to individual peptides from acute HBV patients. Acute HBV patients were stimulated in vitro with 15mer overlapping peptides covering the entire HBV proteome for 10 d and screened for HBV specific T cell responses using 2 dimensional IFN-γ Elispot. Short-term lines were stimulated with 5 µg/ml of each individual peptide identified from the IFN-γ elispot for 5 h and screened using intracellular cytokine staining or CD107a degranulation assay to confirm individual peptide responses. Remaining cells from the 10 d culture were stimulated overnight with confirmed peptides and supernatants were harvested to screen for CXCL-8 production in figure 3B.
(TIF)
