# Supplementary figures and images for: Licensing Virus-Specific T Cells to Secrete the Neutrophil Attracting Chemokine CXCL-8 during Hepatitis B Virus Infection
Source: PLoS One. 2011 Aug 18;6(8):e23330. doi: 10.1371/journal.pone.0023330 (PMC3158071; doi:10.1371/journal.pone.0023330)

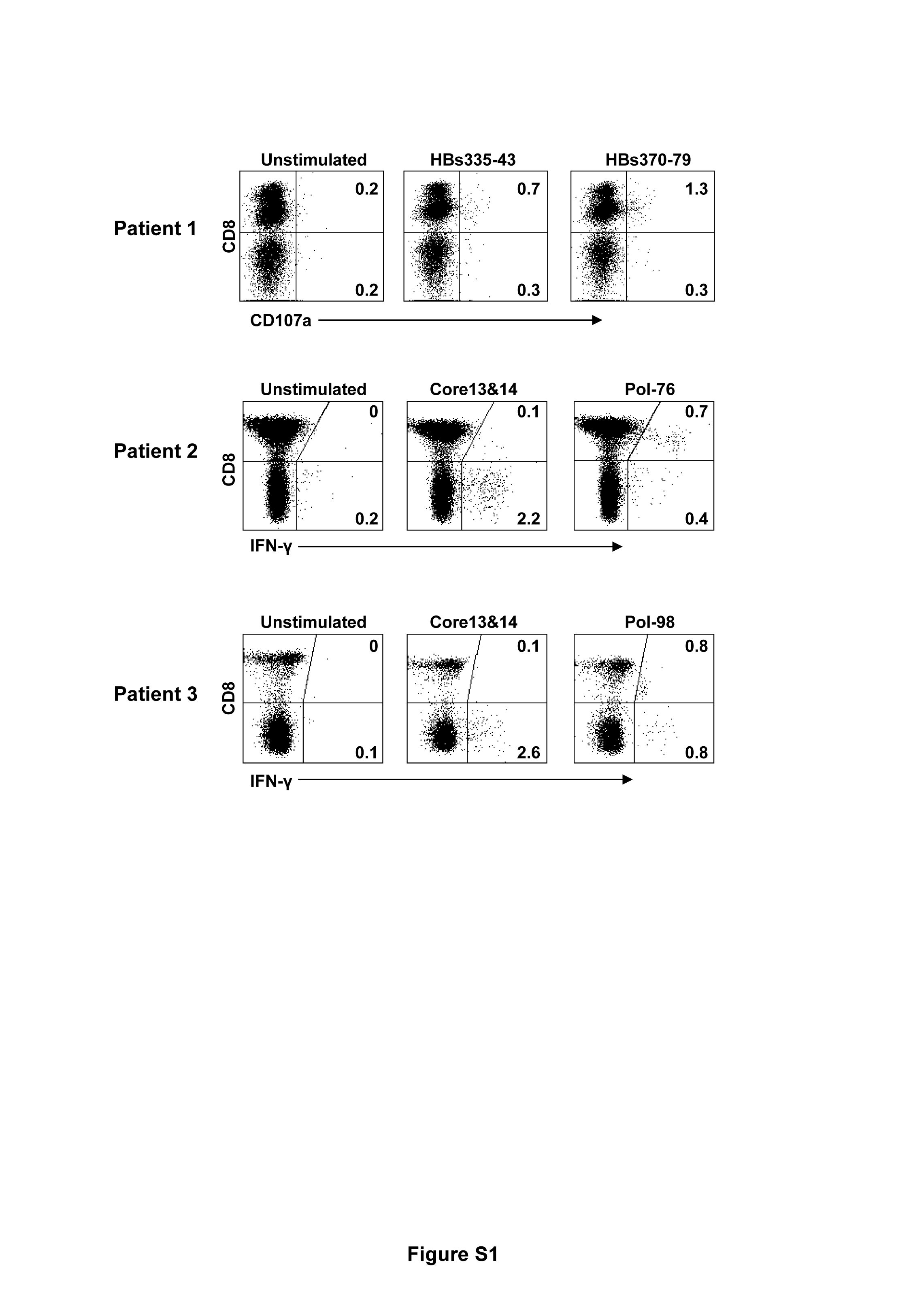

Supplement: Figure S1 — T cell responses to individual peptides from acute HBV patients. Acute HBV patients were stimulated in vitro with 15mer overlapping peptides covering the entire HBV proteome for 10 d and screened for HBV specific T cell responses using 2 dimensional IFN-γ Elispot. Short-term lines were stimulated with 5 µg/ml of each individual peptide identified from the IFN-γ elispot for 5 h and screened using intracellular cytokine staining or CD107a degranulation assay to confirm individual peptide responses. Remaining cells from the 10 d culture were stimulated overnight with confirmed peptides and supernatants were harvested to screen for CXCL-8 production in figure 3B. (TIF) [file pone.0023330.s001.tif]
